# Harmine Induces Adipocyte Thermogenesis through RAC1-MEK-ERK-CHD4 Axis

**DOI:** 10.1038/srep36382

**Published:** 2016-11-02

**Authors:** Tao Nie, Xiaoyan Hui, Liufeng Mao, Baoming Nie, Kuai Li, Wei Sun, Xuefei Gao, Xiaofeng Tang, Yong Xu, Baishan Jiang, Zhengcao Tu, Peng Li, Ke Ding, Weiping Han, Shaoping Zhang, Aimin Xu, Sheng Ding, Pentao Liu, Adam Patterson, Garth Cooper, Donghai Wu

**Affiliations:** 1 Key Laboratory of Regenerative Biology and Guangdong Provincial Key Laboratory of Stem Cell and Regenerative Medicine, Guangzhou Institute of Biomedicine and Health, Chinese Academy of Sciences, Guangzhou, China; 2 State Key Laboratory of Pharmaceutical Biotechnology, The University of Hong Kong, Hong Kong, China; 3 HKU Shenzhen Institute of Research and Innovation, Shenzhen, China; 4 Gladstone Institute of Cardiovascular Disease, Department of Pharmaceutical Chemistry, University of California, San Francisco, CA, USA; 5 Wellcome Trust Sanger Institute, Hinxton, Cambridge, U.K.; 6 Singapore Bioimaging Consortium and Institute of Molecular and Cell Biology, Agency for Science, Technology and Research (A*STAR), Singapore; 7 Maurice Wilkins Centre for Molecular Biodiscovery, School of Biological Sciences, Thomas Building, The University of Auckland, Private Bag 92019, Auckland Mail Centre, Auckland 1142, New Zealand; 8 Department of Pharmacology, University of Oxford, Mansfield Road, Oxford, U.K.; 9 Centre for Advanced Discovery and Experimental Therapeutics, Manchester Biomedical Research Centre, Central Manchester University Hospitals NHS Foundation Trust, Manchester M13 9WL, UK; and the Centre for Endocrinology & Diabetes, Institute of Human Development, The University of Manchester, Manchester Academic Health Sciences Centre, Manchester, U.K.; 10 Joint School of Biological Sciences, Guangzhou Institute of Biomedicine and Health, Guangzhou Medical University, Guangzhou, China.

## Abstract

Harmine is a natural compound possessing insulin-sensitizing effect in *db/db* diabetic mice. However its effect on adipose tissue browning is unknown. Here we reveal that harmine antagonizes high fat diet-induced adiposity. Harmine-treated mice gained less weight on a high fat diet and displayed increased energy expenditure and adipose tissue thermogenesis. *In vitro*, harmine potently induced the expression of thermogenic genes in both brown and white adipocytes, which was largely abolished by inhibition of RAC1/MEK/ERK pathway. Post-transcriptional modification analysis revealed that chromodomain helicase DNA binding protein 4 (CHD4) is a potential downstream target of harmine-mediated ERK activation. CHD4 directly binds the proximal promoter region of *Ucp1,* which is displaced upon treatment of harmine, thereby serving as a negative modulator of *Ucp1*. Thus, here we reveal a new application of harmine in combating obesity *via* this off-target effect in adipocytes.

Obesity, an independent risk factor for diseases ranging from metabolic syndrome to cancer, is becoming epidemic in both developing and developed countries. Efficient ways to increase energy expenditure are urgently needed to combat the escalating occurrence of obesity.

Mammals have two distinct adipose tissues; white adipose tissue and brown adipose tissue. White adipocytes are derived from *Myf5*-negative precursor cells and possess a large unilocular lipid droplet^1^. Brown adipose tissue expresses high levels of uncoupling protein 1 (UCP1) and is thereby responsible for energy expenditure in the form of heat^2^. Recently a third type of adipocytes—beige or brite (brown in white) adipocytes—has been identified in both human and animal models^3^. These UCP1-positive, multilocular brown-like cells are scattered in white adipose tissues and readily induced upon certain stimuli. Conceivably, enhancing the biogenesis and activation of brown or beige adipocytes are potential strategies to combat obesity and its related diseases. Although thermogenesis in brown and white adipose tissue is induced by cold exposure or β3-adrenergic receptor activation^4^,^5^,^6^, more effective approaches to activate brown/beige adipocytes without side effects are still lacking.

Harmine is a naturally occurring harmala alkaloid present in a number of plants and a major component of the psychoactive mixture ayahuasca. It belongs to the beta-carboline family and inhibits dual-specificity tyrosine-regulated kinase-1a (DYRK1A), monoamine oxidase-A (MAO-A), and CDC-like kinases^7^. It has both antitumor and antidepressive functions and protects against type 1 and type 2 diabetes by distinct mechanisms^8^,^9^. In a high-throughput screening for human β-cell mitogenic compounds, harmine emerged as the singular postive hit and was subsequently found to improve islet mass and glycemic control in animal models^10^. Harmine targets white adipose tissue to upregulate proliferator-activated receptor gamma (PPARγ) expression and thus alleviates insulin resistance in *db/db* mice without affecting body weight gain^11^,^12^. However, the effect of harmine and its mechanisms of action are yet to be fully elucidated.

## Results

### Harmine Confers C57BL/6J Mice Resistance to High-Fat Diet–Induced Obesity

**Figure 1:**
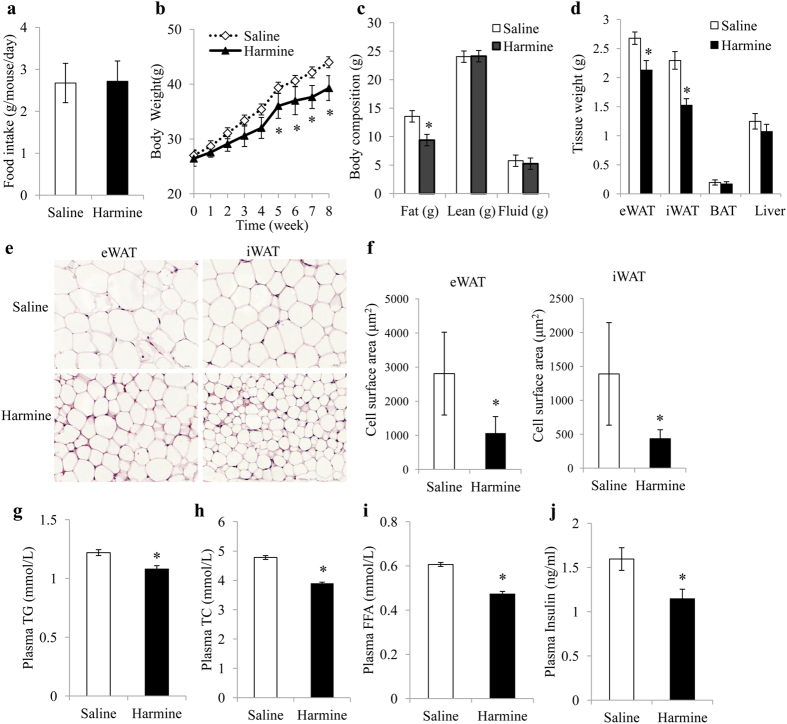
Harmine protects mice against high fat diet induced-obesity. 8-week-old C57BL/6J male mice (n = 9 mice/group) were fed a high fat diet and treated with saline or harmine for 8 weeks. **(a)** Food intake, **(b)** body weight, **(c)** body composition and **(d)** tissue weight. **(e)** Representative H&E staining of epididymal white adipose tissue (eWAT) and inguinal white adipose tissue (iWAT) and **(f)** Cell size. **(g)** Plasma levels of triglycerol (TG), **(h)** total cholesterol (TC), **(i)** free fatty acid (FFA), and **(j)** insulin. Data represent mean ± SEM, *p < 0.05.

To explore the function of harmine in metabolic diseases, 8-week-old C57BL/6J mice were fed with high fat diet and treated with daily saline or harmine (50 mg/kg) for 8 weeks. While total food intake did not differ between the two groups (Fig. 1a), mice receiving harmine treatment gained less weight than the control group (Fig. 1b). The reduction in weight was mainly attributed by the lowered level of adiposity; analysis of body composition by NMR showed that harmine–treated mice had less fat content while other fractions, including lean mass and fluid were largely the same between the two experimental groups (Fig. 1c). Additionally, the inguinal and epididymal white adipose tissues weighed less, whereas the weights of brown adipose tissue and liver were similar between the two groups (Fig. 1d). Further, adipocyte size was smaller in harmine-treated mice (Fig. 1e,f) and harmine was found to reduce plasma levels of free fatty acid, triglycerol, sterol, and insulin (Fig. 1g–j).

### Harmine Induces Thermogenesis of Adipose Tissues *in Vivo*

**Figure 2:**
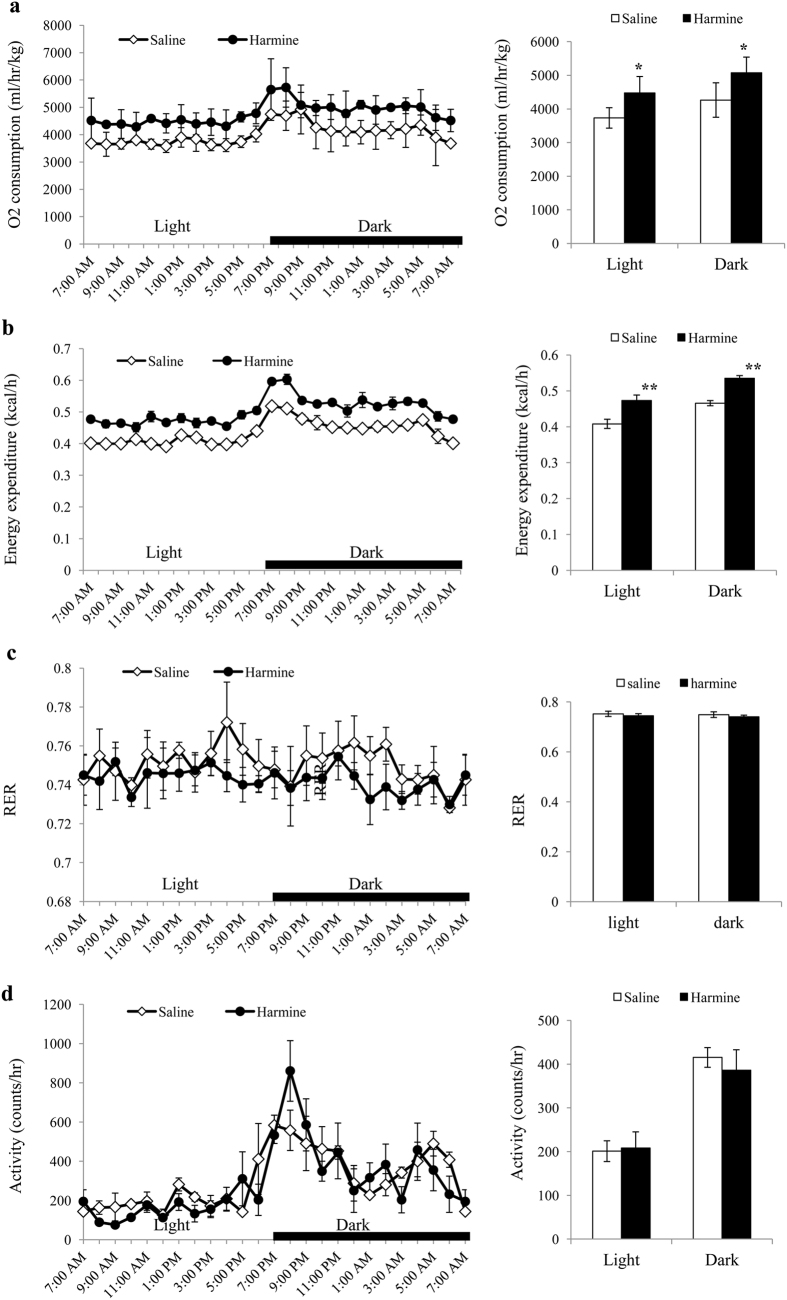
8-week-old C57BL/6J male mice were fed a high fat diet and received daily i.p injection of saline or harmine for 8 weeks (n = 5 mice/group), followed by analysis of **(a)** oxygen consumption, **(b)** heat generation, **(c)** respiratory exchange ratio (RER), and **(d)** activity. Data represent mean ± SEM. *p < 0.05, **p < 0.01.

To investigate the mechanism by which harmine resists diet-induced obesity energy expenditure was monitored in mice by indirect calorimetry. After 8 weeks of treatment, oxygen consumption and energy expenditure during a 12-h light/dark cycle was higher in harmine-treated mice than those in control mice (Fig. 2a,b). Food intake, physical activity, and the respiratory exchange ratio did not differ between the two groups (Fig. 2c,d, Fig. S1).

**Figure 3:**
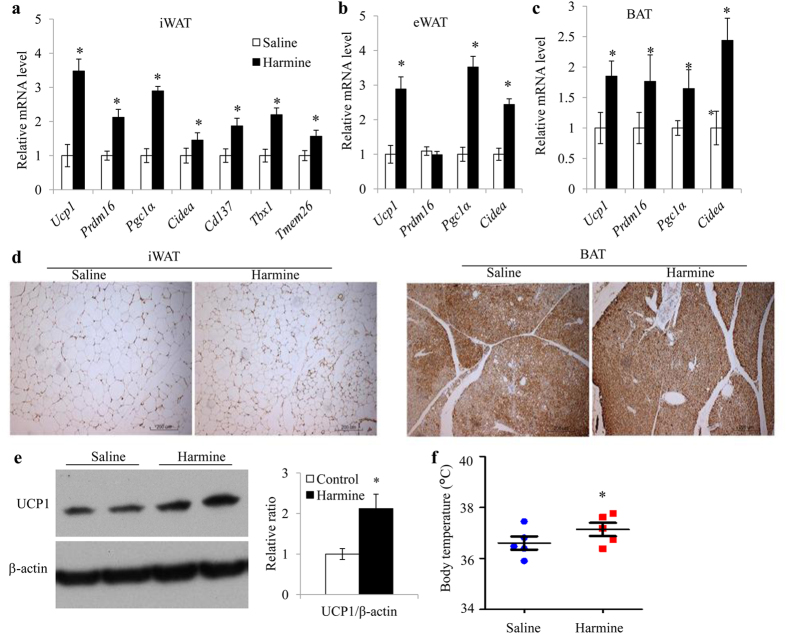
Harmine enhances adipose tissue thermogenesis. 8-week-old C57BL/6J male mice were fed a high fat diet with daily i.p injection of saline or harmine for 8 weeks. **(a–c)** Quantitative PCR analysis of thermogenic genes in **(a)** iWAT, **(b)** eWAT, and **(c)** interscapular brown adipose tissue (BAT) from mice treated with saline or harmine for 8 weeks (n = 4 tissues/group). **(d)** Immunostaining of UCP1 in iWAT (left) and BAT (right). **(e)** Western blot analysis of UCP1 in BAT (left) and densitometric analysis of the relative abundance of UCP1 (right). **(f)** Body temperature of the mice (n = 5 mice/group). Data represent mean ± SEM. *p < 0.05.

Since activation of brown/beige adipocytes is one of the major ways to enhance energy expenditure, we tested whether harmine induces reprogramming to brown/beige in adipose tissues. Real time quantitative PCR revealed that harmine upregulated all of the thermogenic-related genes examined, including *Ucp1, Prdm16, Pgc1α* and *Cidea* in inguinal and brown adipose tissues, and all but *Prdm16* in epididymal adipose tissue (Fig. 3a–c). The upregulation of *Ucp1* expression in inguinal and brown adipose tissues was also confirmed by immunohistochemical staining of UCP1 protein (Fig. 3d,e). Consistent with the elevated levels of thermogenic genes, rectal temperature was higher in harmine-treated mice than controls (Fig. 3f).

### Harmine Induces Adipocyte thermogenesis *in Vitro*

**Figure 4:**
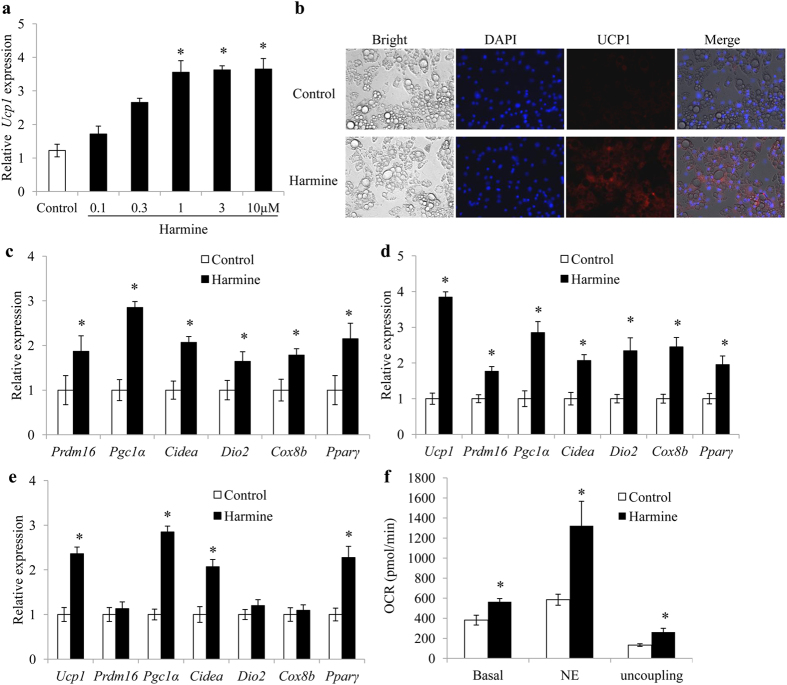
Harmine induces thermogenesis of adipocytes *in vitro*. Inguinal adipose stromal cells were differentiated into adipocytes, treated with different doses of harmine for 24 h, and harvested for qPCR and immunofluorescence analysis. **(a)** *Ucp1* mRNA expression in primary inguinal adipocytes. **(b)** Immunofluorescence staining of adipocytes treated with 1 μM harmine. **(c–e)** Expression of thermogenic genes in **(c)** primary inguinal, (**d)** epididymal, and **(e)** brown adipocytes (n = 5 wells/group). **(f)** OCR in primary inguinal adipocytes treated with saline or harmine (n = 7 well/group). Data represent mean ± SEM, *p < 0.05.

To determine whether harmine directly induces adipocyte thermogenesis, mature adipocytes, differentiated from stromal vascular cells of inguinal adipose tissue, were incubated with harmine at various doses for 48 h. Quantitative real time PCR analysis showed that *Ucp1* expression was readily elevated with increasing doses of harmine with the optimal effect at 1 μM (Fig. 4a). Therefore, in subsequent experiments, 1 μM harmine was used for *in vitro* adipocyte treatment.

Immunofluorescence staining showed that harmine increased the UCP1 protein level (Fig. 4b). In addition, the expressions of other thermogenic genes were also significantly increased by harmine in inguinal primary adipocytes (Fig. 4c). Harmine also induced expression of *Ucp1* and several other thermogenic-related genes in adipocytes derived from brown adipose tissue and epididymal adipose tissue (Fig. 4d,e), suggesting that harmine acts non-selectively on all types of adipocytes *in vitro*. The effect of harmine on adipocyte thermogenesis was further verified by Seahorse bioanalyzer, which indicated that treatment of harmine elevated the oxygen consumption rate at basal, NE-stimulated and uncoupling status (Fig. 4f). However, pretreatment of adipose stromal cells with harmine for 2 days before differentiation did not affect *Ucp1* expression (Fig. S2).

### Harmine-induced browning/beigeing is mediated *via* the RAC1-MEK-ERK Pathway

**Figure 5:**
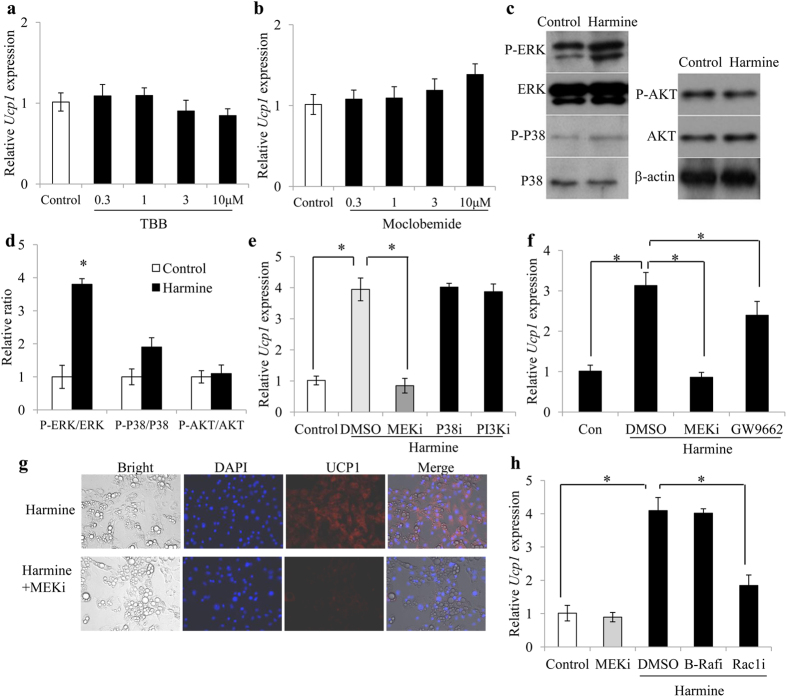
The RAC1-MEK-ERK pathway is essential for harmine-induced thermogenesis. Inguinal adipose stromal cells were differentiated into adipocytes, treated with various chemical inhibitors (1 μM) with or without harmine (1 μM) for 24 h, and then harvested for qPCR and western blot analysis (n = 6 wells/group). **(a,b,e,f)** Quantitative PCR analysis of *Ucp1* expression in the adipocytes treated with harmine and **(a)** DYRK1A inhibitor, **(b)** MAO-A inhibitor, **(e)** inhibitors of MEK (MEKi), P38 (iP38i), and PI3K (PI3Ki), and **(f)** inhibitors of B-Raf (B-Rafi) and RAC1 (Rac1i). **(c)** Representative western blot analysis of ERK, P38, and AKT pathways in primary inguinal adipocytes. **(d)** Densitometric analysis of relative abundance of p-ERK, p-P38, p-AKT proteins as in **(c**). **(g)** Immunofluorescence staining for UCP1 in adipocytes treated with harmine or harmine plus a MEK inhibitor. Data represent mean ± SEM. *p < 0.05.

Harmine is known to inhibit DYRK1A and MAO-A. We therefore tested whether other specific inhibitors of DYRK1A (TBB) and MAO-A (moclobemide) had comparable effects on adipocyte browning. Interestingly, neither compound affected *Ucp1* expression (Fig. 5a,b), suggesting that harmine exerts its thermogenesis activity by targeting other molecules.

Next, we tested the effect of harmine on ERK, p38, and AKT signaling pathways, which are reported to enhance *Ucp1* expression^13^,^14^,^15^. Harmine increased phosphorylation of ERK, while had no detectable effect on the two other kinases examined (Fig. 5c,d). Addition of the MEK inhibitor AZD6244, which specifically inhibits phosphorylation of ERK, completely blocked the induction of *Ucp1* by harmine (Fig. 5e). In contrast, inhibitors against p38 and PI3K (SB202190 and LY294002) failed to block induction of *Ucp1*. Moreover, although previous studies have reported that harmine is capable of inducing PPARγ, the PPARγ inhibitor GW9662 only partially blocked harmine-induced increases in thermogenesis (Fig. 5f). These data indicate that the browning/beiging effects of harmine are mediated through activation of MEK-ERK cascade. The effect of MEKi on harmine induced-UCP1 expression was also corroborated by immunofluorescence analysis (Fig. 5g).

Ras-Raf-MEK-ERK is a classic signaling cascade implicated in cell growth, differentiation and death. Unexpectedly, the Raf inhibitor PLX-4720 did not mitigate harmine*-*induced *Ucp1* expression (Fig. 5h). Intriguingly, inhibition of RAC1 (by Ehop-016), an alternative regulator of ERK pathway, reduced harmine-elicited upregulation of *Ucp1* by 44% (Fig. 5h). We consistently obtained similar results in primary epididymal and brown adipocytes. Notably, the induction of phosphorylation on ERK by harmine was also validated in adipose tissues of mice as well (Fig. S4). These findings demonstrate that harmine induces adipocyte thermogenesis through the RAC1/MEK/ERK pathway.

### CHD4 Is a Potential Target of ERK leading to Increase *Ucp1* Expression

**Figure 6:**
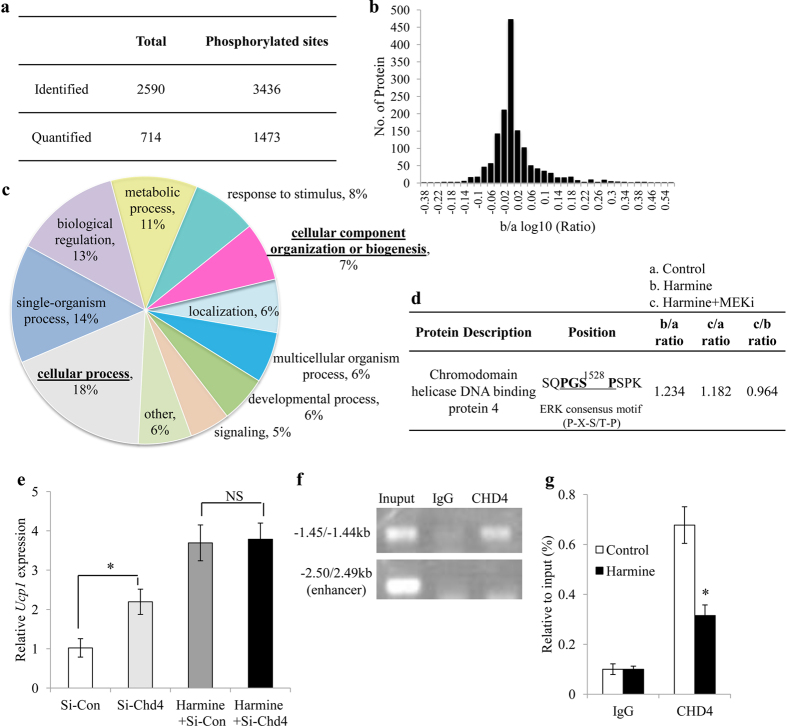
CHD4 negatively regulates harmine-induced *Ucp1* expression. **(a–d)** Inguinal adipocytes treated with DMSO, harmine or harmine plus MEKi were subjected to phosphorylation profiling. **(a,b)** Summary of identified phosphorylation sites and proteins in post-translational modification (PTM) analysis. **(c)** Distribution of phosphorylated proteins by GO terms. **(d)** Ratio of phosphorylated proteins in treated groups and controls. PTM analysis shows that CHD4 is a potential target of ERK. **(e)** *Chd4* knockdown promotes *Ucp1* expression in adipocytes and does not have a synergistic effect on thermogenesis with harmine (n = 5 wells/group). Si-Con, control siRNA; Si-Chd4, siRNA targeting *Chd4*. **(f)** ChIP assay with primers amplifying a ~100-bp fragment at −2.5 kb or at −1.4 kb. **(g)** Quantification of the bands in panel **(f)** (n = 3 wells/group). Data represent mean ± SEM. *p < 0.05.

Next, we examined the molecular events linking harmine-induced ERK activation and thermogenic gene expression. To this end, primary inguinal adipocytes were treated with DMSO, harmine, or harmine plus MEK inhibitor and the protein lysates were subjected to proteomic analysis with phosphorylation profiling. A total of 2590 phospho-proteins with 3436 phosphorylation sites were identified (Fig. 6a), of which 1473 phosphorylation sites in 714 proteins were further analyzed and quantified (Fig. 6a,b). To identify the link between ERK substrates and upregulation of *Ucp1* expression, we focused on proteins involved in cellular process or transcriptional regulation or biogenesis, as suggested by Gene Ontology analysis (Fig. 6c). Chromodomain helicase DNA binding protein 4 (CHD4) appeared to be a potential candidate, as its phosphorylation was enhanced by harmine and blocked by MEK inhibitor. Bioinformatic analysis revealed that CHD4 has a P-X-S-P motif, a preferred phosphorylation site of ERK (Fig. 6d).

**Figure 7:**
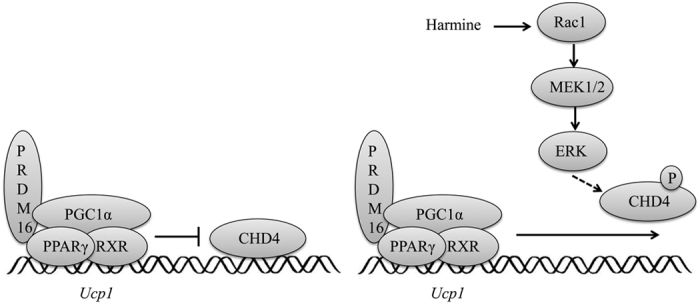
Schematic diagram of the thermogenesis mechanism by harmine. CDH4 serves as a transcription repressor of *Ucp1* by binding on its proximal promoter region. Harmine induces the RAC1/Mek1/2/ERK signaling cascade, which in turn phosphorylates CHD4. The phosphorylated CHD4 is thereby released from the *Ucp1* promoter and activates the transcription of *Ucp1* gene.

To verify the involvement of CHD4 in *Ucp1* expression, siRNA targeting *Chd4 were* transfected into primary inguinal adipocytes. The result demonstrated that knock-down of endogenous Chd4 led to increased mRNA level of *Ucp1* at basal level (Fig. 6e, Fig. S5). Interestingly, the induction of *Ucp1* by Chd4 knock-down was abolished when adipocytes were treated with harmine (Fig. 6e), indicating that CHD4 and harmine act in the same signaling cascade. ChIP assay showed that CHD4 directly binds to the –1.4 kb region in the promoter of *Ucp1* but not to its enhancer region (Fig. 6f), and the binding was significantly decreased by harmine (Fig. 6g). These results demonstrate that CHD4 serves as a cis-transcriptional suppressor of *Ucp1* and harmine transactivates *Ucp1* at least partially through its attenuation on the transcriptional suppressive effects of CHD4 (Fig. 7).

## Discussion

New drugs are urgently needed to treat obesity and related metabolic diseases. Several chemical compounds and cytokines appear to reduce obesity at least in part by inducing adipose tissue browning/beiging and thermogenesis^16^,^17^,^18^,^19^. In the current study we found that anti-tumor, anti-depressive, and anti-β cell death compound harmine, is also a potent agent to promote the thermogenesis of adipocytes and thus prevent diet-induced obesity. Although we cannot rule out that harmine recruits beige adipocytes *in vivo*, in our screening system it is clear that harmine is capable of converting mature white adipocytes into beige adipocytes *in vitro*. In a study of *db/db* mice, harmine improved insulin sensitivity, but did not affect adiposity or reduce body weight after 4 weeks of treatment^11^. It is possible that *db/db* mice are more resistant than C57BL/6J mice to the thermogenesis and weight-loss effects of harmine.

Harmine is an active ingredient in ayahuasca, which has been used for centuries in the Amazon and Orinoco River basins for medicinal and ceremonial purposes. Ayahuasca is believed to alleviate obesity and other health problems, although the mechanism is incompletely understood^20^. We found that harmine at the estimated human equivalent dose suppresses body weight gain in obese mice, an effect partially achieved by enhancing adipose tissue thermogenesis. It should be noted that the thermogenic effect of harmine in adipocytes are independent of its known targets, including MAO-A and DYRK1, since unrelated compounds blocking these two enzymes do not mimic the effects of harmine. These findings may be useful for developing harmine-based drug leads to treat obesity. Future drug development efforts should be directed at decreasing its ability to cross the blood-brain barrier and enhancing its specificity for adipose tissues.

The direct target of harmine that mediates adipose tissue thermogenesis and weight reduction is not known. MAO-A, a major inhibitory target of harmine, catalyzes the oxidative deamination of amines such as dopamine, norepinephrine, and serotonin. Norepinephrine potently induces adipocyte thermogenesis, raising the possibility that the *in vivo* effect of harmine reflects elevated norepinephrine levels in adipose tissues. However, in a clinical study of 18 adult men, MAO activity in abdominal subcutaneous adipose tissue was 50% lower in obese subjects than in lean healthy controls^21^. Moreover, other MAO inhibitors, which have been widely used to treat depression, have not been reported to have weight-lowering effects^22^. Thus, the anti-obesity effects of harmine may not be mediated through MAOs.

Harmine also inhibits DYRK1A, which interacts with Raf to activate the ERK pathway^23^. Harmine inactivates the ERK pathway in neuronal cells and inhibits differentiation of osteoclasts without affecting the ERK pathway^24^. In contrast, our study showed that harmine significantly enhanced the ERK signaling cascade in adipocytes, and a Raf inhibitor failed to mitigate its effect on thermogenesis. Evidently, harmine targets multiple targets and its pharmacological effect in different tissues and cell types may be determined by the relative level of different targets in specific tissues. Studies with tissue-specific knockout mice are needed to further elucidate the molecular target and mechanisms of action of harmine.

Our study provides strong evidence that RAC1/MEK/ERK have a predominant role in enhancing the thermogenic features in adipocytes. Irisin, another potential thermogenic hormone, also upregulates *Ucp1* expression, a function that was abolished by ERK inhibition^25^. However, the effect of the ERK pathway on *Ucp1* expression is still somewhat controversial^15^,^26^. The detailed mechanism whereby activation of ERK leads to *Ucp1* upregulation is unclear. *In vitro*, ERK signaling pathway promoted PPARγ^27^, which transactivates *Ucp1* through a 220-bp PPARγ response element in the *Ucp1* enhancer^28^. On the other hand, although studies by Tontonoz’s group and us both found that harmine increases PPARγ expression in primary adipocytes, inhibition of PPARγ only partially blocked the effect of harmine on thermogenesis, which is in sharp contrast to the finding that MEK inhibitor completely inhibited the activity of harmine. Thus, PPARγ might partially mediate downstream molecular events of harmine-ERK signaling cascade to promote *Ucp1* expression. It should also be noted that direct addition of harmine to a biochemical kinase assay with recombinant MEK protein did not lead to its phosphorylation, suggesting that harmine does not directly active MEK. The direct target of harmine is to be identified in future studies.

The incomplete inhibition of harmine by GW9662 prompted us to perform phosphorylation profiling, which identified CHD4 as a potential target of ERK. Unlike PPARγ, CHD4 is a major component of the nucleosome remodeling and deacetylase complex and interacts with HDAC1 and HDAC2 to serve as an epigenetic transcriptional repressor^29^. Our ChIP analysis showed that CHD4 binds to the proximal region of *Ucp1* promoter, instead of the upstream enhancer region where positive transactivation factors reside. Thus, CHD4 and PPARγ may work synergistically to facilitate harmine-induced expression of *Ucp1*.

In conclusion, we found that harmine promotes the thermogenesis of adipocytes and thus has potential as a treatment for obesity. A better understanding of the molecular targets and mechanisms will be useful for developing new therapies for obesity and related metabolic diseases.

## Methods

### Mice

Eight-week-old male C57BL/6J mice (The Jackson Laboratory) were maintained on a 12-h light/12-h dark cycle at 23 °C. The mice were fed a high fat diet (D12492, Research Diet) for 8 weeks and given daily injection (i.p.) of saline or harmine (TCI, 442-51-3, >98%(T), 50 mg/kg body weight) at 10:00 am. The diet provides 21.9 kJ/g: 60% of energy from fat, 20% from protein, and 20% from carbohydrate. Food intake was measured daily and mice were weighed weekly. Rectal temperature was measured at 2:00 pm at the end of the experiment. Animal experiments were approved by the Animal Care and Use Committee of Guangzhou Institute of Biomedicine and Health (GIBH), Chinese Academy of Sciences. All animal experiments were conducted in accordance with the GIBH Guide for the Care and Use of Laboratory Animals.

### Isolation of Adipocytes and Stromal Vascular Cells from Adipose Tissues

Adipose tissues were dissected from C57BL/6J mice, rinsed in phosphate-buffered saline, minced, and digested for 40 min at 37 °C in 0.1% (w/v) type I collagenase solution (Sigma) in D-Hanks buffer. The digested tissue was filtered through a 250-μm nylon mesh and centrifuged at 800 × g for 3 min. The sediment was resuspended in Dulbecco’s modified Eagle’s medium (DMEM, GIBCO) with 10% fetal bovine serum (Hyclone). Two days after reaching confluence (day 0), the cells were induced to differentiate into adipocytes in medium containing 5 μg/ml insulin (Sigma), 1 μM dexamethasone (Sigma), 0.5 mM isobutylmethylxanthine (Calbiochem), and 1 μM rosiglitazone (Sigma). Two days later, the medium was replaced with DMEM supplemented with 10% fetal bovine serum, 5 μg/ml insulin, and 1 μM rosiglitazone, and the cells were cultured for 6 days.

### Cell Treatment

Differentiated adipocytes were treated in DMEM plus 10% FBS, with addition of 1 μM harmine or DMSO (as vehicle control) in the presence or absence of 1 μM inhibitors for 24 h. After that, the cells were harvested for real time qPCR analysis. TBB (sc-202830) was purchased from Santa Cruz Biotechnology; moclobemide, ZD6244, SB202190, LY294002 and GW9662 were from Selleck.

### siRNA Transfection

siRNA oligonucleotides were designed and synthesized by RiboBio Company (Guangzhou, China). On day 8 of differentiation, siRNA (20 nmol) was transfected into the primary adipocytes with Lipofectamine2000 (Invitrogen) according to the manufacturer’s instructions. Eight hours after transfection, the medium was replaced with normal culture medium. The adipocytes were treated with drugs 48 h later as indicated and harvested for analysis.

### RNA Extraction and Quantitative PCR

Total RNA was isolated with Trizol (Invitrogen), and first-strand cDNA was synthesized with Superscript III Reverse Transcriptase (Invitrogen) with 0.5 μg of RNA as the template for each reaction. mRNA levels were quantified under optimized conditions with SYBR Premix Ex Taq (Takara Bio) following the manufacturer’s instructions. 18S ribosomal RNA was used as the reference gene.

### Seahorse analysis

The oxygen consumption rate (OCR) of cells was analyzed using the XF^e^24 Seahorse bioanalyzer. One day prior to analysis, cells were treated with harmine (1μM) or DMSO. 24 h later, the cells were equilibrated in sodium carbondioxide-free DMEM for 1 h in CO2 free incubator. The following drugs were sequentially loaded to each wells to measure the basal, NE-stimulated, uncoupling-related OCR: NE (10 μM), antimycin A (5 μM), FCCP (5 μM), rotenone (3 μM) plus oligomycin (5 μM).

### Post-translational Modification Analysis

Mouse stromal cells from inguinal fat were differentiated into mature adipocytes and treated with harmine, harmine plus MEK inhibitor, or DMSO (mock control) for 24 h. The cells were washed three times with cold phosphate-buffered saline and flash frozen at −80 °C. Proteomic analysis of phosphorylation profiling was done at Jingjie PTM Biolabs (Hangzhou, China).

### Histology and Immunohistochemistry

Brown adipose tissue and inguinal and epididymal adipose tissues were fixed in 4% formaldehyde overnight at room temperature, embedded in paraffin, and sectioned (5 μm) by microtome. The slides were deparaffinized, rehydrated, and stained with hematoxylin and eosin (Sigma) by a standard protocol. Alternatively, the sections were stained with anti-UCP1 (ab23841, Abcam; 1:200) and developed with SIGMAFAST DAB with Metal Enhancer (Sigma). Sections were examined by light microscopy (Motic BA600) and photographed with Moticam Pro 285A. Photomicrographs were scanned with an Abaton Scan 300/Color scanner.

Indirect Calorimetry and Calculated Energy Expenditure Whole-body oxygen consumption was measured with an open-circuit indirect calorimetry system with automatic temperature and light controls (Comprehensive Lab Animal Monitoring System, Columbus Instruments). Mice had ad libitum access to chow and water in respiration chambers, and data were recorded for 48 h, including 24 h of acclimatization. Energy expenditure was calculated as recommended by the manufacturer.

### Chromotain Immunoprecipitation

Chromotain immunoprecipitation (ChIP) was done with the Pierce ChIP Kit (Agarose 26156), following the manufacturer’s protocol. Briefly, mature adipocytes derived from inguinal stromal cells were exposed to 1% formaldehyde at room temperature for 10 min to induce DNA cross-linking and then lysed and digested with MNase for 10 min on ice. The cell lysate supernatant was incubated with IgG or CHD4 antibody (Abcam, ab72418) at 4 °C overnight and then with ChIP Grade Protein A/G Plus Agarose for 1 h. Bound DNA was isolated and recovered as instructed by the manufacturer. The primer pairs used to detect the *Ucp1* promoter were GTCAGTCACCCTTGACCACA and TAGGGGTGAGGCTGATATCCC (enhancer region, −2.5 kb) and ACACACACCACTGACTGCTC and AGCGTGCACACTAAGCAAGA (proximal region, −1.4 kb).

### Statistical Analysis

Data are expressed as means ± SEM. ANOVA and unpaired, two-tailed *t* tests were used for most comparisons. Tukey’s HDS test was used for body weight gain and indirect calorimetry data. P < 0.05 was considered significant.

## Additional Information

**How to cite this article**: Nie, T. *et al*. Harmine Induces Adipocyte Thermogenesis through RAC1-MEK-ERK-CHD4 Axis. *Sci. Rep.* **6**, 36382; doi: 10.1038/srep36382 (2016).

**Publisher’s note:** Springer Nature remains neutral with regard to jurisdictional claims in published maps and institutional affiliations.

## Supplementary Material

Supplementary Information
