# Supplementary material for: Harmine Induces Adipocyte Thermogenesis through RAC1-MEK-ERK-CHD4 Axis
Source: Sci Rep. 2016 Nov 2;6:36382. doi: 10.1038/srep36382 (PMC5090989; doi:10.1038/srep36382)
Supplement: Supplementary Information [file srep36382-s1.pdf]

Harmine Induces Adipocyte Thermogenesis through RAC1-MEK-ERK-CHD4 Axis

Tao Nie,[1*] Xiaoyan Hui,[2, 3*¶] Liufeng Mao,[1] Baoming Nie,[4] Kuai Li,[1] Wei Sun,[1] Xuefei Gao,[5] Xiaofeng Tang,[1] Yong Xu,[1] Baishan Jiang,[1] Zhengcao Tu,[1] Peng Li,[1] Ke Ding,[1] Weiping Han,[6] Shaoping Zhang,[7] Aimin Xu,[2, 3] Sheng Ding,[4] Pentao Liu,[5] Adam Patterson,[7] Garth Cooper,[7,8,9] and Donghai Wu[1¶]

[1]Key Laboratory of Regenerative Biology and Guangdong Provincial Key Laboratory of Stem Cell and Regenerative Medicine, Guangzhou Institute of Biomedicine and Health, Chinese Academy of Sciences, Guangzhou, China

[2]State Key Laboratory of Pharmaceutical Biotechnology, The University of Hong Kong, Hong Kong, China

[3]Department of Medicine, The University of Hong Kong, Hong Kong, Hong Kong, China

[4]Gladstone Institute of Cardiovascular Disease, Department of Pharmaceutical Chemistry, University of California, San Francisco, CA

[5]Wellcome Trust Sanger Institute, Hinxton, Cambridge, U.K.

[6]Singapore Bioimaging Consortium and Institute of Molecular and Cell Biology, Agency for Science, Technology and Research (A*STAR), Singapore

[7]Maurice Wilkins Centre for Molecular Biodiscovery, School of Biological Sciences, Thomas Building, The University of Auckland, Private Bag 92019, Auckland Mail Centre, Auckland 1142, New Zealand.

[8]Department of Pharmacology, University of Oxford, Mansfield Road, Oxford, U.K.

[9]Centre for Advanced Discovery and Experimental Therapeutics, Manchester Biomedical Research Centre, Central Manchester University Hospitals NHS Foundation Trust, Manchester M13 9WL, UK; and the Centre for Endocrinology & Diabetes, Institute of Human Development, The University of Manchester, Manchester Academic Health Sciences Centre, Manchester, U.K.

[*]These authors contributed equally.

[¶]Corresponding authors: Xiaoyan Hui, hannahui@hku.hk, Donghai Wu, wu_donghai@gibh.ac.cn

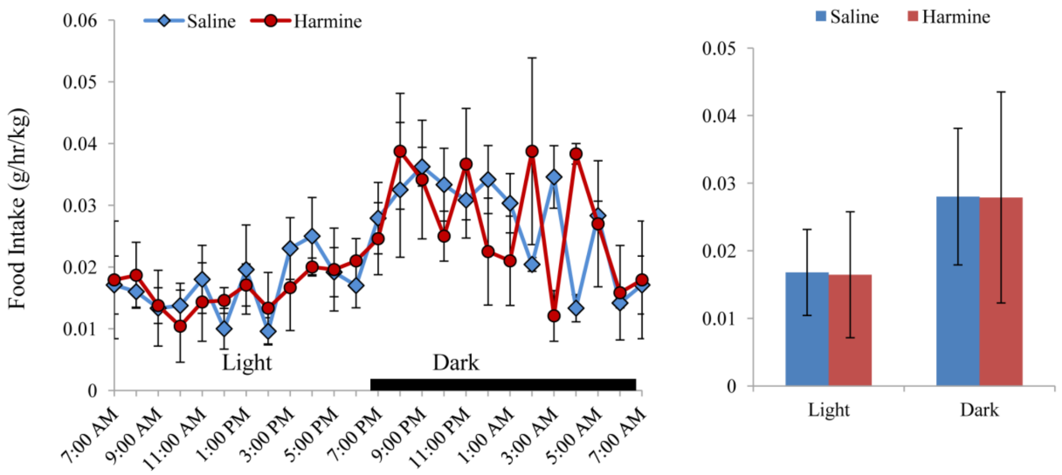

Figure S1. Food consumption in CLAMS study of mice fed with harmine or saline as control (n=5 mice).

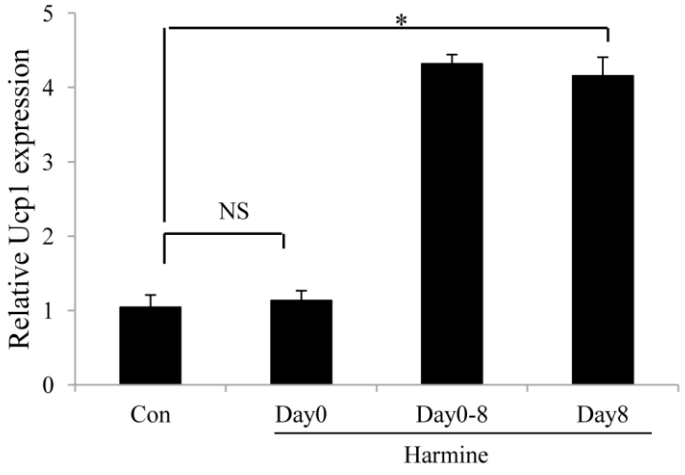

Figure S2 Harmine cannot convert inguinal adipose stromal cells into brite/beige precusor cells. Data represent mean ± SEM, n=3 independent experiments, 3 wells for each experiment, *p<0.05.

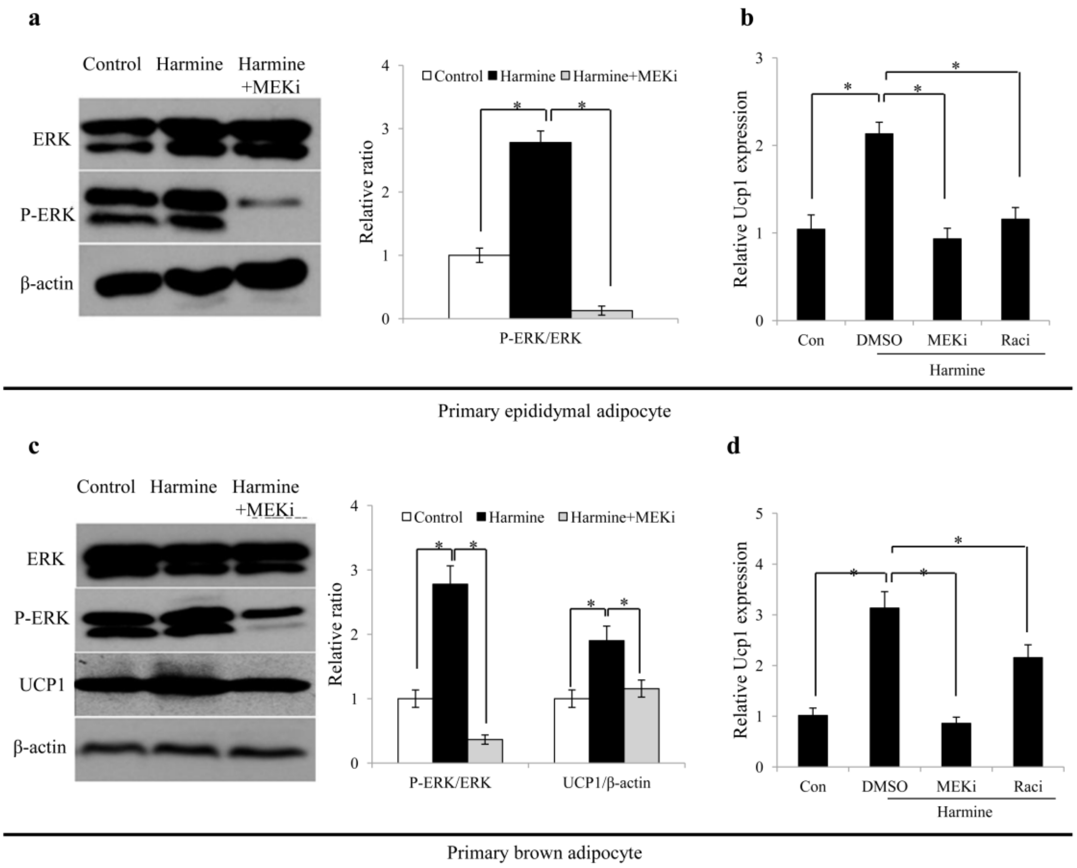

Figure S3 Harmine induces browning in primary epididymal and brown adipocytes.

a. Left: Western blot results of ERK pathway in primary epididymal adipocytes treated with harmine or harmine plus MEK inhibitor. Right: Densitometric analysis for relative abundance of p-ERK/t-ERK proteins. b. *Ucp1* mRNA expression in primary epididymal adipocytes. c. Left: Western blot results of ERK pathway in primary brown adipocytes treated with harmine or harmine plus MEK inhibitor. Right: Densitometric analysis for relative abundance of p-ERK/t-ERK proteins. d. *Ucp1* mRNA expression in primary brown adipocytes. Data represent mean ± SEM, n=3 independent experiments, 3 wells for each experiment,*$p<0.05$.

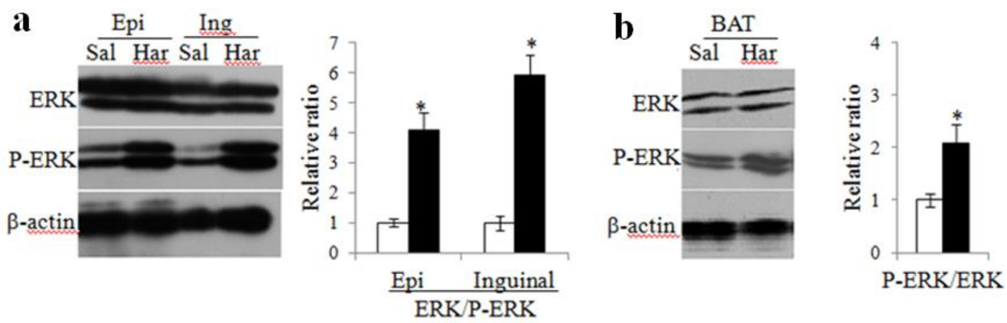

Figure S4 Harmine induces phosphorylation of ERK in adipose tissues *in vivo*. C57/BL6J male mice were fed with HFD with daily i.p. injection of saline or harmine for 8 weeks. The epididymal (Epi), inguinal (ing) WAT and BAT were isolated for Western blot analysis. Left: Representative images of western blot results of ERK pathway in epididymal and inguinal (a) WATs and BAT (b). Right: Densitometric analysis for relative abundance of p-ERK/t-ERK proteins. Data represent mean ± SEM, n=3 independent experiments, 5 samples for each experiment, $^*p<0.05$.

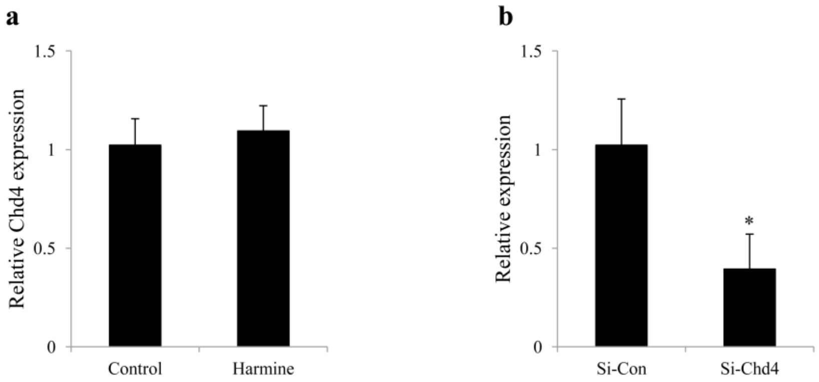

Figure S5 a. Harmine did not alter the mRNA expression of *Chd4* in primary inguinal adipocyte. b. *Chd4* siRNA effectively silenced the mRNA expression of *Chd4* compared to control siRNA (n=3 wells). Data represent mean ± SEM, n=3 independent experiments.
